# Successful large gene augmentation of *USH2A* with non-viral episomal vectors

**DOI:** 10.1016/j.ymthe.2023.06.012

**Published:** 2023-06-19

**Authors:** Maria Toms, Lyes Toualbi, Patrick V. Almeida, Richard Harbottle, Mariya Moosajee

**Affiliations:** 1 Development, Ageing, and Disease, UCL Institute of Ophthalmology, London EC1V 9EL, UK; 2 Ocular Genomics and Therapeutics, The Francis Crick Institute, London NW1 1AT, UK; 3 DNA Vector Research, German Cancer Research Center (DKFZ), 69120 Heidelberg, Germany; 4 Department of Genetics, Moorfields Eye Hospital, NHS Foundation Trust, London EC1V 2PD, UK

**Keywords:** *USH2A*, Usher syndrome, retinitis pigmentosa, non-viral gene therapy, scaffold matrix attachment region

## Abstract

*USH2A* mutations are a common cause of autosomal recessive retinitis pigmentosa (RP) and Usher syndrome, for which there are currently no approved treatments. Gene augmentation is a valuable therapeutic strategy for treating many inherited retinal diseases; however, conventional adeno-associated virus (AAV) gene therapy cannot accommodate cDNAs exceeding 4.7 kb, such as the 15.6-kb-long *USH2A* coding sequence. In the present study, we adopted an alternative strategy to successfully generate scaffold/matrix attachment region (S/MAR) DNA plasmid vectors containing the full-length human *USH2A* coding sequence, a GFP reporter gene, and a ubiquitous promoter (CMV or CAG), reaching a size of approximately 23 kb. We assessed the vectors in transfected HEK293 cells and *USH2A* patient-derived dermal fibroblasts in addition to *ush2a*^*u507*^ zebrafish microinjected with the vector at the one-cell stage. pS/MAR-USH2A vectors drove persistent transgene expression in patient fibroblasts with restoration of usherin. Twelve months of GFP expression was detected in the photoreceptor cells, with rescue of Usher 2 complex localization in the photoreceptors of *ush2a*^*u507*^ zebrafish retinas injected with pS/MAR-USH2A. To our knowledge, this is the first reported vector that can be used to express full-length usherin with functional rescue. S/MAR DNA vectors have shown promise as a novel non-viral retinal gene therapy, warranting further translational development.

## Graphical abstract

**Figure:**
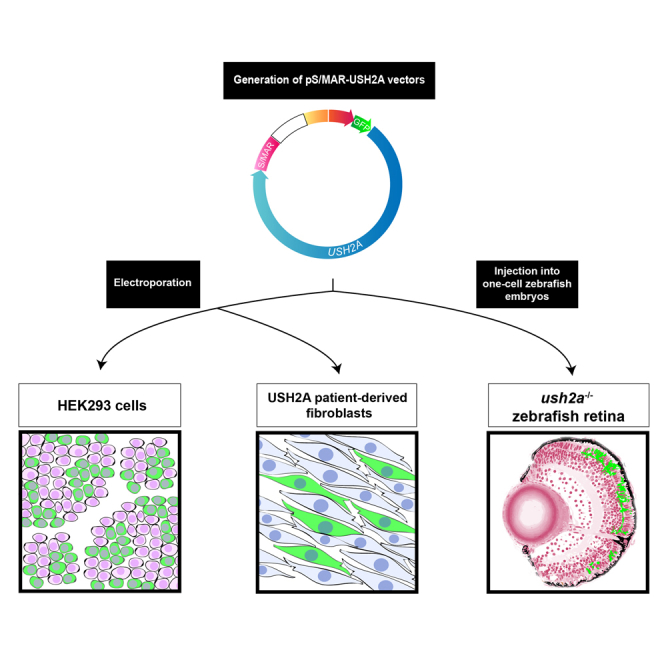

## Teaser

Moosajee and colleagues have generated episomal plasmid vectors containing the coding region of the very large *USH2A* gene, a common cause of the retinal disease retinitis pigmentosa. These vectors were assessed in cellular and zebrafish models of *USH2A*, showing promise as a novel non-viral retinal gene therapy.

## Introduction

Mutations in the *USH2A* gene (MIM: 608400) cause autosomal recessive retinitis pigmentosa (RP) (MIM: 613809) or Usher syndrome type IIA (MIM: 276901), a disorder resulting in congenital sensorineural hearing loss and RP.^1^^,^^2^^,^^3^ RP is a degenerative retinal disease involving initial loss of rod photoreceptors, causing night blindness and loss of peripheral vision, eventually leading to cone photoreceptor cell death in later life, affecting central vision and color perception. *USH2A* mutations are estimated to cause up to 23% of non-syndromic RP cases^4^ and around 80% of Usher syndrome type II cases,^5^ representing a significant cause of inherited retinal disease (IRD) in the human population.

*USH2A* is a very large, 72-exon gene located on chromosome 1q41; the full-length transcript has a 15,606-bp coding region, producing a 5,202-amino-acid transmembrane protein known as usherin (isoform b). In addition, a short secreted isoform (a) 1,546 amino acids in size is expressed in various tissues, but the full-length protein is thought to be the predominant form of usherin in the retina and developing inner ear.^6^^,^^7^ In the inner ear, usherin localizes to the transient stereociliary ankle links of the developing hair cells, allowing correct post-natal formation of the stereociliary hair bundle.^8^^,^^9^ In the retina, the protein localizes to the periciliary membrane at the connecting cilium of the photoreceptors, where it forms a complex with the other two Usher syndrome 2 proteins, adhesion G protein-coupled receptor V1 (ADGRV1) and whirlin.^7^^,^^10^^,^^11^^,^^12^ Although its exact role in the photoreceptors is still unclear, studies in *Xenopus*,^10^^,^^13^ mouse,^7^^,^^10^^,^^14^^,^^15^ and zebrafish^16^ suggest that usherin and its associated complex may be involved in protein transport and autophagy. A recent study in the macaque retina demonstrated that Usher proteins establish links between the inner and outer segments of the photoreceptors, first formed by Usher type II proteins at the very base of the cilium and then above by Usher type I proteins, indicating a possible role in mechanotransduction.^17^

While several therapies are under investigation for *USH2A* retinopathy, such as small-molecule drugs,^18^^,^^19^ CRISPR-Cas9-based gene editing,^20^^,^^21^^,^^22^ and antisense oligonucleotides,^23^^,^^24^ there is currently no approved treatment. Furthermore, many therapeutic strategies in development are allele specific and therefore not suitable for all patients. Gene augmentation therapy involves providing a healthy copy of the defective disease-causing gene, typically using viruses to carry the required cDNA into the patients’ tissues. It has been an attractive option for treating IRDs because of the size, accessibility, and immune privilege of the eye, and voretigene neparvovec has become the first approved adeno-associated virus (AAV)-based retinal gene therapy for patients with biallelic *RPE65* retinopathy.^25^ However, the *USH2A* gene is too large for single AAV vectors, which have a limited gene size carrying capacity of less than 5 kb. In addition, therapeutic use of viruses carries the risk of triggering potentially harmful inflammatory responses in the patients’ ocular tissues, which may lead to sight loss and reduced efficacy.^26^

Considering these disadvantages, non-viral alternatives are of great interest for retinal gene therapy, with one strategy being use of a plasmid vector system with potentially unlimited cloning capacity that incorporates a DNA motif known as the scaffold/matrix attachment region (S/MAR). S/MARs are 33- to 61,755-bp-long AT-rich genomic sequences at which the chromatin anchors to the nuclear matrix proteins during interphase, a function thought to be involved in gene regulation.^27^^,^^28^ When incorporated into plasmid DNA vectors, S/MARs have been shown to promote episomal maintenance and mitotic stability and produce persistent gene expression *in vitro* and *in vivo*.^27^ In the *Rpe65*^*−/−*^ mouse retinal pigment epithelium (RPE), nanoparticles complexing S/MAR vectors (VMD2-hRPE65-S/MAR) were able to produce RPE65 and EGFP expression up to 15 months^29^ and 2 years^30^ post subretinal injection, respectively, and to improve electroretinogram (ERG) responses in treated mice. In a recent study, polyethylene glycol (PEG)-(1-aminoethyl)iminobis[N-(oleoylcysteinyl-1-amino-ethyl)propionamide] (ECO)/pGRK1-ABCA4-S/MAR nanoparticles were generated, which consisted of an 11.6-kb plasmid vector containing full-length *ABCA4* and an S/MAR, complexed with the amino lipid ECO modified by PEGylation.^31^ Multiple subretinal administrations of the nanoparticle in *Abca4*^*−/−*^ mice generated long-term gene expression and a substantial reduction in A2E accumulation.

In the present study, we aimed to use a non-viral gene therapy strategy to deliver full-length human *USH2A* to cellular and zebrafish models. We cloned the *USH2A* coding sequence into plasmid vectors containing an S/MAR sequence and a GFP reporter gene with either a CAG or CMV promoter. *USH2A*^*−/−*^ patient-derived dermal fibroblasts were transfected with the pS/MAR-USH2A vectors, producing GFP and usherin protein expression. Additionally, long-term GFP expression was detected in the photoreceptors of wild-type and *ush2a* knockout zebrafish (*ush2a*^*u507*^) injected with the pS/MAR-USH2A vector at the one-cell stage, with rescue of Usher 2 protein localization observed in the mutant zebrafish.

## Results

### Inserting the full-length human *USH2A* cDNA into pS/MAR vectors

**Figure 1:**
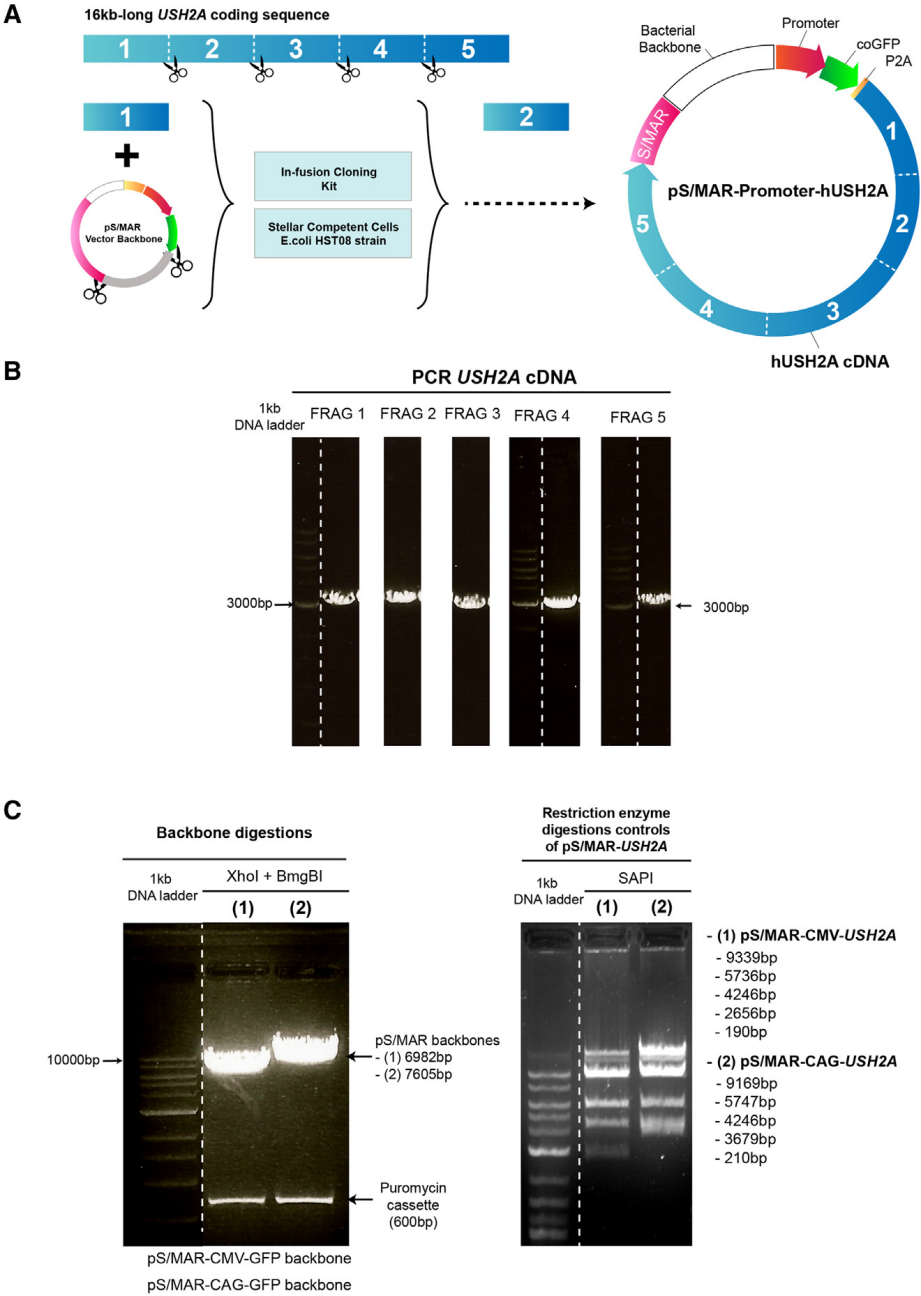
Generation of pS/MAR-USH2A vectors (A) pS/MAR-USH2A vectors were generated by inserting the full *USH2A* coding sequence (16 kb) into the backbone in 5 fragments. The vectors contained a CAG or CMV promoter and *P. plumata* GFP sequence 5′ of the *USH2A* sequence and an S/MAR sequence at the 3′ end. The P2A sequence encodes a self-cleaving peptide. (B) PCR was performed to amplify each *USH2A* fragment. (C) pS/MAR backbone digestion cut out the puromycin cassette and restriction enzyme digestion controls of the pS/MAR-USH2A vectors using SAPI.

Because of its large size, the 15,609-bp *USH2A* cDNA sequence was amplified into five fragments that were inserted piece by piece into the S/MAR plasmid backbones, as described in Figure 1. PCRs were performed to amplify each fragment of *USH2A* cDNA (∼3,000 bp), and 5 cloning steps were successively carried out to insert each into the vector backbones. Restriction enzyme digestion with SAPI and Sanger sequencing of the full insert were performed as quality controls. The two vectors generated either had ubiquitous CAG (pS/MAR-CAG-USH2A) or CMV (pS/MAR-CMV-USH2A) promoters. The vectors also contained the green fluorescent protein (GFP) gene from the copepod species *Pontellina plumata* (also known as copGFP). pS/MAR-CAG-USH2A and pS/MAR-CMV-USH2A were 21.2 kb and 22.2 kb in size, respectively.

### Overexpression of *USH2A* in HEK293 cells and patient-derived dermal fibroblasts

**Figure 2:**
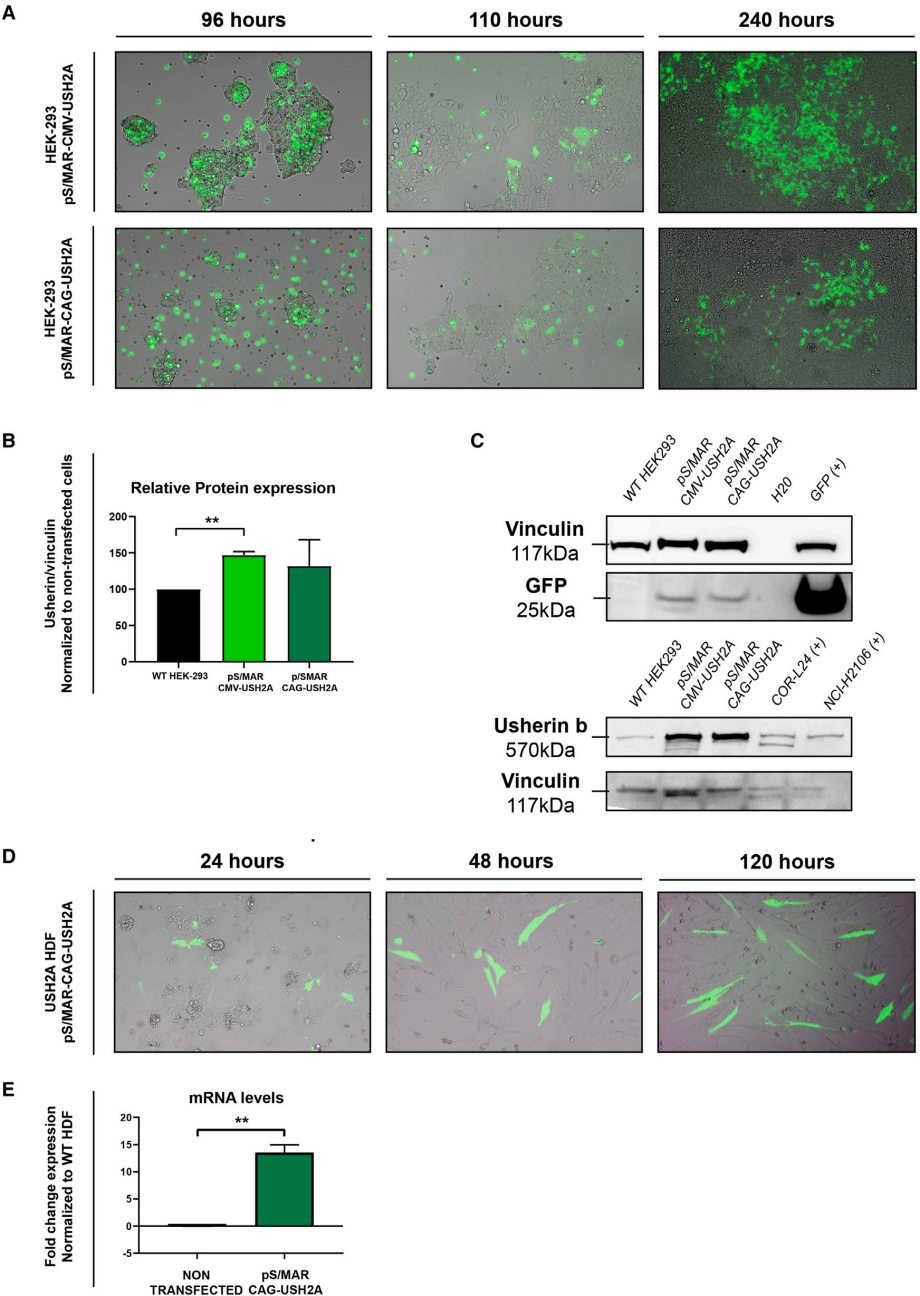
Transfection of HEK293 cells and *USH2A* patient-derived human dermal fibroblasts (A) Fluorescence microscopy images showing GFP expression in HEK293 cells electroporated with pS/MAR-CMV-USH2A and S/MAR-CAG-USH2A vectors at 96, 110, and 240 h post transfection. (B and C) Western blot analysis of usherin expression was performed in non-transfected HEK293 cells and pS/MAR-CMV-USH2A- and pS/MAR-CAG-USH2A-transfected HEK293 cells. GFP was also detected, and vinculin was used as a control. Two cancer lines, COR-L24 and NCI-H2106, were used as positive controls for the usherin protein band. Full blot images for usherin bands are available in the supplemental information. (D) Fluorescence microscopy images showing GFP expression in *USH2A* patient-derived human dermal fibroblasts electroporated with the pS/MAR-CAG-USH2A vector at 24, 48, and 120 h post transfection. (E) Fold change gene expression of *USH2A* in non-transfected or pS/MAR-CAG-USH2A-transfected *USH2A* patient fibroblasts, normalized to non-transfected WT fibroblasts. ∗∗p < 0.01.

Because of the large size of the pS/MAR-USH2A vectors generated in this study, electroporation was required to aid cell transfection. Electroporation of HEK293 cells with pS/MAR-CMV-USH2A and pS/MAR-CAG-USH2A was conducted, and GFP expression was monitored by fluorescent microscopy 96, 110, and 240 h post transfection (Figure 2A). Western blotting of HEK293 cells electroporated with pS/MAR-CMV-USH2A and pS/MAR-CAG-USH2A detected expression of GFP and usherin isoform b (570 kDa) (Figure 2B). Usherin was also detected in non-transfected HEK293 cells at lower levels. Expression of usherin in transfected HEK293 cells was 147.1% ± 3.9% (CMV vector) and 131.9% ± 29.5% (CAG vector) compared with non-transfected cells (n = 3). As positive controls for the usherin band, we used two different cell lines reported to express *USH2A*: CORL24, a small lung cancer cell line, and NCI-H2106, a large-cell lung carcinoma (https://depmap.org/portal/).

**Figure 3:**
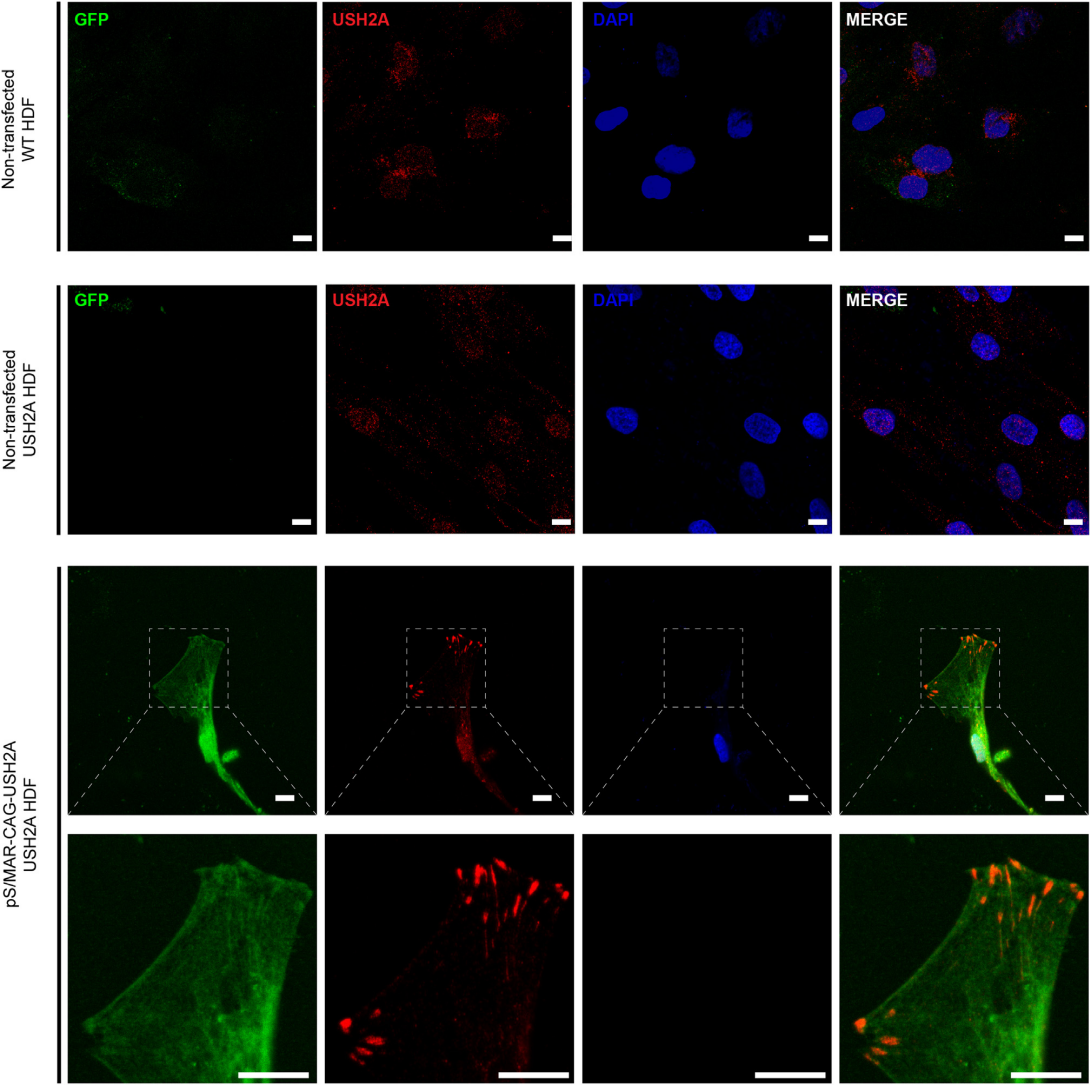
Over-expression of usherin in dermal fibroblasts transfected with pS/MAR-USH2A *USH2A* patient-derived dermal fibroblasts, non-transfected or transfected with pS/MAR-CAG-USH2A, were immunostained with anti-usherin (red). GFP (green) was also detected, and DAPI nuclei stain (blue) was used. Scale bars, 10 μm.

For transfection of *USH2A* patient-derived dermal fibroblasts, pS/MAR-CAG-USH2A was used because this was found to generate more persistent GFP expression (Figure 2C). qRT-PCR of *USH2A* confirmed significantly higher levels of expression (13.5 ± 1.2-fold) in transfected *USH2A* fibroblasts compared with non-transfected cells (n = 3, p > 0.01) (Figure 2D). Immunostaining of wild-type and *USH2A* fibroblasts was performed to detect usherin expression (Figure 3). In fibroblasts transfected with pS/MAR-USH2A, a specific and novel expression pattern of usherin could be observed compared with background levels of the protein or non-specific staining observed in the control fibroblasts. GFP expression was also detected in these transfected fibroblasts. Immunostaining of 42-week-old wild-type retinal organoids was performed to validate the specificity of the usherin antibody (Figure S2).

### Generation of *USH2A* expression in zebrafish

**Figure 4:**
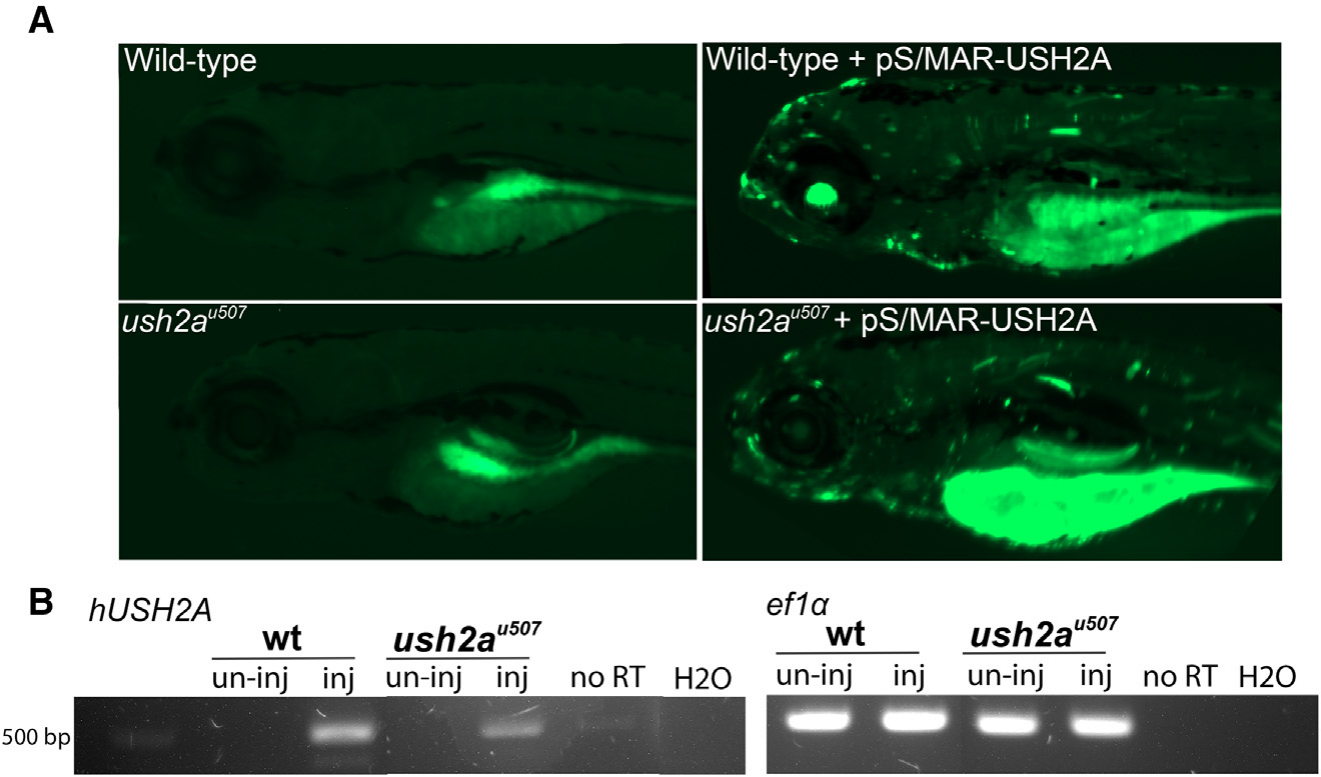
pS/MAR-USH2A vector expression in zebrafish (A) WT and *ush2a*^*u507*^ zebrafish were micro-injected with pS/MAR-CMV-USH2A at the one-cell stage. GFP expression was detected in injected WT and *ush2a*^*u507*^ larvae at 5 dpf. (B) RT-PCR analysis detected human *USH2A* (*hUSH2A*) expression in injected (inj) zebrafish and not in un-injected (un-inj) larvae. *ef1a* gene expression was used as a control. No reverse transcription (no RT) and water (H_2_O) controls were performed. The full gel is available in the supplemental information.

To investigate pS/MAR-USH2A vector expression in an *in vivo* model, wild-type (WT) and *ush2a*^*u507*^ zebrafish were injected with pS/MAR-CMV-USH2A at the one-cell stage. This promoter was found to drive higher expression in zebrafish compared with CAG (Figure S3). GFP expression in the injected zebrafish embryos could be seen by ∼6 h post injection. At 5 days post fertilization (dpf), mosaic expression of GFP was observed throughout the injected WT and *ush2a*^*u507*^ zebrafish larvae (Figure 4A). RT-PCR analysis was used to detect the expression of human *USH2A* mRNA in injected zebrafish (Figure 4B). Total DNA was also isolated from the injected zebrafish and subjected to rolling circle amplification, which confirmed the episomal status of the DNA nanovectors, as described previously^32^ (data not shown).

**Figure 5:**
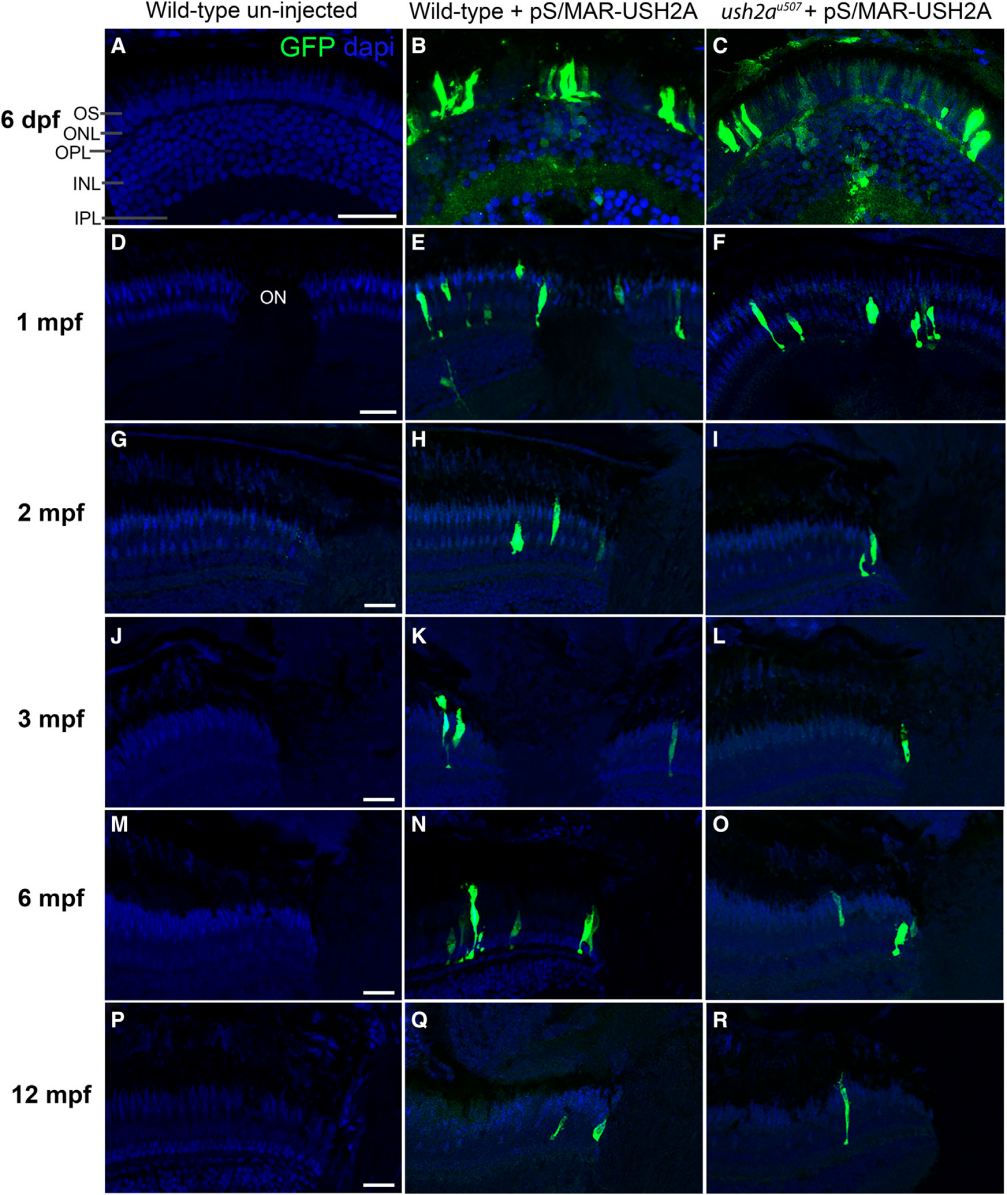
Long-term expression of pS/MAR-USH2A in the zebrafish retina GFP expression was observed in the retina of WT and *ush2a*^*u507*^ zebrafish injected with pS/MAR-CMV-USH2A at the one-cell stage. Anti-turboGFP was used to aid GFP detection on retinal sections at ages 6 days post-fertilization (dpf) (A–C), 1 month post-fertilization (mpf) (D–F), 2 mpf (G–I), 3 mpf (J–L), 6 mpf (M–O), and 12 mpf (P–R). Sections were counterstained with DAPI nucleic acid stain (blue). OS, photoreceptor outer segment; ONL, outer nuclear layer; OPL, outer plexiform layer; INL, inner nuclear layer; IPL, inner plexiform layer; ON, optic nerve. Scale bars, 25 μm.

Retinal cryosections from injected zebrafish were immunostained with anti-GFP to aid detection of pS/MAR vector-generated GFP expression (Figure 5). GFP was observed predominantly in the photoreceptors of injected WT and *ush2a*^*u507*^ zebrafish at 6 dpf but could also be detected in the nuclear and plexiform layers of the retina (Figures 5B and 5C). Several time points between 6 dpf and 12 months post fertilization (mpf) were examined for GFP expression, which was detected in the retinas of injected zebrafish at every age investigated but mostly limited to photoreceptors in the proximity of the optic nerve from 1 mpf and older. To assess whether the size of the vector may affect retinal expression levels, we micro-injected WT zebrafish with an equivalent vector containing the GFP coding sequence only (pS/MAR-CMV-copGFP, 7.2 kb) and examined GFP localization in the retina at 1 mpf (Figure S5). GFP expression was found to be comparable with that produced by the *USH2A* vector and mostly confined to the photoreceptors.

**Figure 6:**
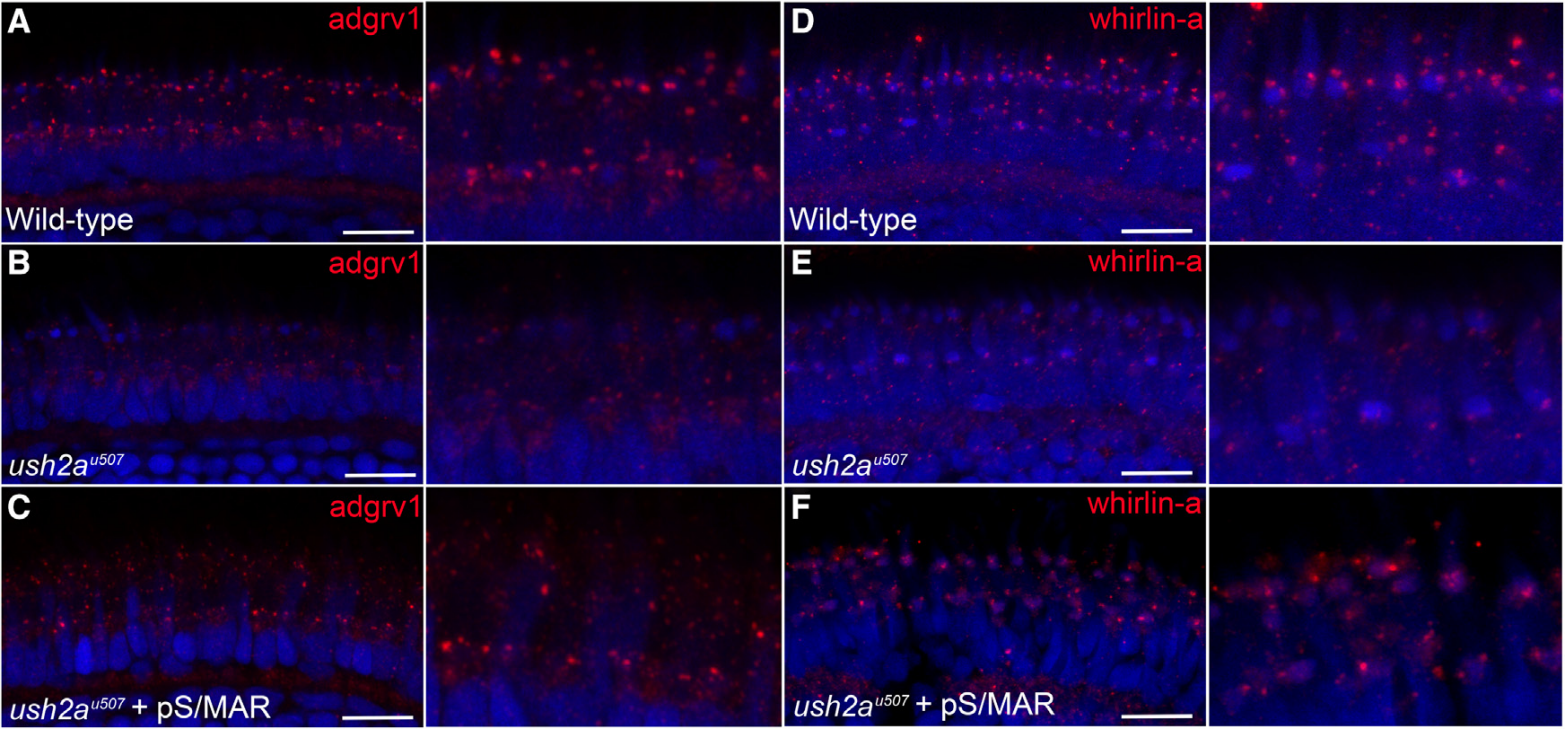
Partial rescue of the Usher 2 protein complex in pS/MAR-USH2A-injected *ush2a*^*u507*^ zebrafish Retinal sections from zebrafish at 6 dpf were immunostained with antibodies for two Usher 2 complex proteins: adgrv1 (A–C) and whirlin-a (D–F). Specific punctate expression of adgrv1 (red, A) and whirlin-a (red, D) was detected in the WT photoreceptors, while both proteins were mislocalized in the *ush2a*^*u507*^ retina (B and E). In *ush2a*^*u507*^ zebrafish injected with pS/MAR-CMV-USH2A at the one-cell stage, specific adgrv1 and whirlin-a expression could be observed in some photoreceptors (C and F). Scale bar, 10 μm.

It was not possible to detect human usherin expression on the zebrafish retinal sections because the antibodies tested were not optimal because of high levels of non-specific staining. Alternatively, to investigate whether functional usherin protein was being produced by the S/MAR vector, the localization of the Usher 2 protein complex was examined in the zebrafish retina. adgrv1, whirlin-a, and whirlin-b are interacting partners of usherin, forming a complex at the periciliary membrane of the photoreceptor-connecting cilium.^11^ adgrv1, whirlin-a, and whirlin-b were detected in the photoreceptors of WT zebrafish at 6 dpf (Figures 6A, 6D, and S6A). In the *ush2a*^*u507*^ retina, loss of usherin disrupted the complex, as noted by reduced expression of all three proteins (Figures 6B, 6E, and S6B). In the photoreceptors of *ush2a*^*u507*^ that were injected with pS/MAR-USH2A, restoration of Usher 2 protein expression was observed (Figures 6C, 6F, and S6C). It was not possible to co-localize GFP because of the heating antigen retrieval step required for the Usher 2 antibodies; however, the retinal sections used for this experiment were selected because of high levels of photoreceptor GFP expression detected in adjacent sections.

## Discussion

Biallelic pathogenic variants in *USH2A* contribute significantly to the proportion of patients with IRDs worldwide, causing Usher syndrome type IIA and non-syndromic RP. Although there have been substantial advancements in virus-based gene therapies for IRDs in recent years, limits on gene size carrying capacity and reports of inflammatory responses in treated patients^26^ highlight the need for alternative gene augmentation strategies. In the present study, we successfully cloned the full 15.6-kb *USH2A* cDNA into non-viral S/MAR vectors and generated persistent transgene expression *in vitro* and *in vivo*.

Initially, we transfected pS/MAR-USH2A vectors using electroporation into two human cell lines: HEK293 cells and patient dermal fibroblasts. Persistent GFP expression was detected in both cell lines. Usherin protein (large isoform, 570 kDa) was also detected by western blot analysis of HEK293 cells. To our knowledge, this is the first study reporting successful overexpression of human usherin in *in vitro* and *in vivo* models of Usher syndrome IIA. Yu et al.^33^ previously overexpressed full-length mouse usherin with its signal peptide in different cell lines for structural modeling. The function or localization of the full-length protein was not examined. However, they transfected COS-7 cells with an extracellular portion of usherin (F19–F32) and showed homogeneous well-targeted expression at the membrane of the cells. Similarly, Samanta et al.^19^ investigated endogenous expression of usherin in fibroblasts and found usherin to be localized to the cell membrane in healthy donor cells, which was disrupted in untreated patient-derived fibroblasts. In our study, exogenous usherin produced by p/SMAR-USH2A appears to localize to a specific part of the cell, which, we propose, may be the focal adhesion. In line with this hypothesis, a recent study showed expression of Usher II protein ADGRV1 at the focal adhesions of several cell lines, such as hTERT-RPE1, mouse embryonic fibroblasts, and mouse astrocytes, showing a pattern similar to the usherin overexpression reported in our study.^34^

To assess pS/MAR-USH2A expression *in vivo*, the previously CRISPR-Cas9-generated *ush2a*^*u507*^ zebrafish was used, which shows loss of usherin protein and retinal defects, including reduced ERG responses and progressive rod cell degeneration.^16^ When pS/MAR-USH2A was injected at the one-cell stage, GFP was detected in the developing embryos, including the retinal photoreceptors. Photoreceptor expression of GFP was detected up to 12 mpf (the oldest time point assessed), although fluorescent photoreceptors became sparser with age because of the continued growth of the zebrafish retina from the ciliary marginal zone. There was a mosaic expression pattern of GFP throughout the zebrafish larval body, which showed preferential expression in certain cell types and was likely silenced by certain lineages during development. Assessing different promoters may improve the range of expression, or using a mammalian animal model may be more suitable. In addition, direct injection of the S/MAR DNA into the zebrafish eye may improve expression.

Usherin forms a complex with ADGRV1 and whirlin at the photoreceptor-connecting cilium, and proper localization of this complex is disrupted when any of the proteins are defective.^11^^,^^12^^,^^35^ Because of genome duplication, zebrafish possess two whirlin proteins: whirlin-a and whirlin-b. Consistent with previous studies including other *ush2a* zebrafish mutants,^11^ loss of adgrv1, whirlin-a, and whirlin-b was observed in *ush2a*^*u507*^ photoreceptors. Promisingly, zebrafish injected with pS/MAR-USH2A showed rescue of these three Usher 2 proteins in the retina, indicating that the vector was producing functional usherin capable of restoring the Usher 2 complex.

To date, there is a limited number of studies using non-viral vectors for therapeutic delivery of large genes. To our knowledge, the largest plasmid vector delivered *in vivo* was 20.2 kb; this luciferase plasmid with a CMV promoter was transfected into the mouse liver using PEG-substituted lysine 30-mers (CK30PEG).^36^ For retinal work, vectors of either approximately 12 kb,^31^^,^^37^^,^^38^ 14 kb,^39^ or 16 kb^38^ in size containing the *ABCA4* coding sequence have been used successfully to transfect the *Abca4*^*−/−*^ mouse retina, producing months of protein expression and improvements in the disease phenotype compared with untreated mice. All studies packaged the plasmids into nanoparticles, CK30PEG^39^ or multifunctional pH-sensitive amino lipid ECO nanoparticles,^31^^,^^37^^,^^38^ to increase the transfection rate. Low retinal transfection efficiency, particularly in photoreceptors, remains a challenge for non-viral therapy. In this study, we used either electroporation or intracellular injection to aid transfection; however, in future work, using a molecular vehicle for safe and efficient intraocular injection will be necessary for higher-order animal models and clinical translation, especially considering the size of the *USH2A* vectors. Some nanoparticles, including ECO,^40^^,^^41^ can be modified with ligands to aid cell targeting, making them an attractive option.

A further consideration for non-viral therapy is loss of transgene expression over time because of the silencing or loss of the vector. Although the S/MAR element is thought to protect against transgene silencing,^42^ the presence of unmethylated CpG dinucleotides and bacterial sequences in the plasmid backbone may contribute to immune detection and silencing. This issue has been addressed by producing minimally sized S/MAR vectors, known as nanoS/MARs, where the bacterial backbone, which includes the origin of replication and antibiotic resistance gene, was removed.^43^ In a *SMAD4* knockout pancreatic cancer cell line, the nanoS/MAR was found to drive higher transgene expression and elicit minimal changes in gene expression related to immune pathways. The nanovectors have also been shown to be effective in generating transgenic T cells without causing molecular damage.^32^ In addition to reducing the likelihood of silencing, removal of these unneeded elements from the *USH2A* vector would have the added benefit of size reduction.

Other strategies under investigation for therapeutic delivery of large genes to the retina include use of dual or triple-AAV vectors to expand the carrying capacity, which has been used for *MYO7A*,^44^^,^^45^^,^^46^^,^^47^ *ABCA4*,^46^^,^^47^ and *ALMS1*.^48^ However, the transduction efficiency was limited compared with single vectors. For instance, in pig photoreceptors, the transduction levels of dual AAVs were about 2- to 3-fold lower than those obtained with a single AAV vector of normal size.^47^ Furthermore, the capacities were still not increased enough to accommodate a cDNA the size of *USH2A*. Intein-mediated protein *trans*-splicing has been used to improve the transduction efficiency when using multiple AAVs and was used to restore *ABCA4* and *CEP290* expression in the corresponding mouse mutants.^49^ Although promising, using this method to deliver *USH2A* to the retina would require more than 5 AAV particles. Alternatively, adenoviral vectors, such as helper-dependent adenoviruses, have a sufficient cargo capacity (up to 36 kb) to carry the full *USH2A* cDNA but are highly immunogenic.^50^ With all virus-based strategies discussed, there is a considerable risk of eliciting harmful inflammatory responses in the patient, such as sight-threatening reactions, which have been reported in several clinical and preclinical studies, including those for *CHM* and *RPE65* therapies.^26^

There are several strategies in development for treating *USH2A*-related disease; these include antisense oligonucleotide-induced exon-skipping for mutations in exon 13 via intravitreal injection^24^ (ClinicalTrial.gov: NCT03780257), CRISPR-Cas9 gene editing,^20^^,^^21^^,^^22^ and translational readthrough-inducing drugs (TRIDs).^18^^,^^19^ These therapies show much promise but are limited to patients with specific alleles or mutation types. In contrast, the benefit of gene augmentation (either viral or non-viral) is its suitability for all *USH2A* patients with a loss of functional protein.

In conclusion, we successfully cloned the *USH2A* cDNA into non-viral episomal vectors and demonstrated the potential of these vectors in replacing large protein expression in different *in vivo* and *in vitro* disease models. Although further investigation showing efficiency in the mammalian retina is necessary, developing a safe and effective non-viral large gene therapy will be hugely beneficial to a significant proportion of retinal disease patients requiring functional genes that exceed the AAV limit.

## Materials and methods

### S/MAR vector generation

All vector modifications on pS/MAR were performed using the In-Fusion cloning strategy (Takara Bio) following the manufacturer’s instructions. The *USH2A* cDNA was kindly provided by Prof. Luk Vandenberghe (Harvard Medical School). To clone the *USH2A* cDNA into the S/MAR vector backbones, it was initially amplified by PCR using Phusion High-Fidelity DNA Polymerase (Thermo Fisher Scientific, F530S). Five fragments of PCR-amplified *USH2A* cDNA of similar size (∼3,000 bp) were produced using CloneAmp HiFi PCR Premix (Clontech, 639298) for further in-fusion cloning using the In Fusion HD Cloning Kit (Clontech, 639649), fragment by fragment. Each primer set was designed to introduce 15-bp homologous overhangs for efficient recombination plus an enzymatic restriction site. First, 1–3 μg of vector backbone was digested with 1–3 units of XhoI and BmgBI for 1 h at 37°C. For the recombination reaction, 100 ng of vector and 50 ng of the insert were mixed with the In-Fusion mix and incubated at 50°C for 15 min. Stellar competent cells (Takara Bio) were used for transformation. The cloning was repeated five times to get the complete *USH2A* cDNA insert into the S/MAR vector backbone. Finally, restriction enzyme digestion with SAPI and Sanger sequencing of the full insert were performed for quality control.

### Cell culture

WT and *USH2A*^*−/−*^ patient-derived human dermal fibroblasts were obtained from skin biopsies. Targeted *USH2A* sequence analysis of the patient cells confirmed compound heterozygosity for the c.2299del p.(Glu767Serfs∗21) and c.3187_3188del p.(Gln1063Serfs∗15) variants. Cells were cultured in DMEM high glucose (Gibco, 41966029) supplemented with 15% fetal bovine serum (FBS; Gibco, 10500064) and 1% Pen/Strep (Gibco, 15140122). After reaching 80% confluency, the cells were passaged using TrypLE Express Enzyme (Gibco, 12605028) and the medium was changed twice a week. HEK293 cells were cultured under the same conditions but with the cell culture medium supplemented with 10% FBS. The cells were maintained at 37°C under a 5% CO_2_/95% air atmosphere, 20% oxygen tension, and 80%–85% of humidity.

Transfection of human dermal fibroblasts and HEK293 cells was carried out using the Neon Transfection System 100 μL Kit (MPK10025). Briefly, cells were dissociated using TrypLE Express (Gibco, 12605028) and counted using a Countess II Automated cell counter. For each transfection, 1 million cells were resuspended in 100 μL buffer R, mixed with 5–10 μg of vector DNA, and electroporated with the following parameters: 1,650 V, 10 ms, 3 pulses for human dermal fibroblasts and 1,450 V, 10 ms, 2 pulses for HEK293 cells. GFP expression was monitored by fluorescent microscopy. Medium was changed 24 h after electroporation.

### Zebrafish husbandry and microinjection

Zebrafish (WT AB and *ush2a*^*u507*^) were bred and maintained according to local UCL and UK Home Office regulations for the care and use of laboratory animals under the Animals Scientific Procedures Act at the UCL Bloomsbury campus and The Francis Crick Institute zebrafish facilities. The UCL Animal Welfare and Ethical Review Body approved all procedures for experimental protocols, in addition to the UK Home Office (license PPL PC916FDE7). All approved standard protocols followed the guidelines of the ARVO Statement for the Use of Animals in Ophthalmic and Vision Research Ethics. Zebrafish were microinjected with 25 pg of vector DNA into the cell at the one-cell stage of development.

### RT-PCR and qRT-PCR

Total RNA was extracted from cells using the RNeasy mini kit or from pools of 10 whole zebrafish embryos using the RNeasy FFPE kit (QIAGEN, Hilden, Germany). cDNA was synthesized from 1 μg of RNA using the Superscript II First Strand cDNA Synthesis Kit (Thermo Fisher Scientific) according to the manufacturer’s instructions. For RT-PCR with zebrafish samples, amplification of a 514-bp human *USH2A* sequence was performed using MyTaq DNA polymerase (Bioline). *ef1α* primers were used as a control. All primer information is provided in Table S1. The following PCR cycle conditions were used: incubation at 95°C for 1 min for initial denaturation, followed by 35 cycles of 95°C for 15 s (denaturation); annealing temperature for 15 s and 72°C for 15 s (extension). For qRT-PCR analysis of cell samples, *USH2A* transcript levels were analyzed using SYBR Green MasterMix (Thermo Fisher Scientific) on a StepOne real-time PCR system (Applied Biosystems, Thermo Fisher Scientific) under standard cycling conditions. All samples were assayed in triplicate.

### Western blot

Cells were washed twice with cold PBS and lysed in RIPA cell lysis buffer supplemented with 1× protease and phosphatase inhibitor cocktail. The cell lysate was centrifuged at 4°C for 20 min at 13,000 rpm. The protein supernatant was collected, and the concentration was measured using the BCA Protein Assay Kit. For each sample, 40 μg of protein was boiled at 95°C for 5 min with NuPAGE sample buffer and reducing agent. Gel electrophoresis was performed using NuPAGE Tris acetate SDS running buffer gel, which was then wet transferred onto an Immuno-Blot polyvinylidene fluoride (PVDF) membrane for 16 h at 25 V and 4°C. The membrane was incubated in blocking buffer solution (5% dry milk/PBS-0.1% Tween [0.1% PBST]) for 1 h before overnight incubation at 4°C under agitation with an anti-usherin antibody (kindly provided by Prof. Aziz El-Amraoui, Institut Pasteur) diluted 1:1,000 in blocking buffer and anti-vinculin antibody as a loading control (1:2,000, sc-25336, Thermo Fisher Scientific). The membrane was then washed 3 times with 0.1% PBST and further incubated with horseradish peroxidase (HRP)-coupled secondary antibodies (P044701-2 or P044801-2, Agilent Technologies) diluted in blocking solution for 1 h at room temperature under agitation. The membrane was washed 3 times before chemiluminescence detection by the Clarity ECL Western Blotting Substrate (Bio-Rad, 1705061) and the ChemiDoc MP Imaging system. ImageJ was used to analyze the results.

### Vector isolation and episomal analysis

For detection of episomally maintained DNA vectors, the TempliPhi Amplification Kit (GE Healthcare) was used, following the manufacturer’s protocol. Total DNA was extracted with the Wizard Genomic DNA Purification Kit (Promega) and quantified using a NanoDrop 2000c (Thermo Fisher Scientific) spectrophotometer. 20 ng of total DNA was used for each reaction. The samples were first denatured at 95°C for 3 min and quickly transferred into ice. The master mix provided in the kit was added, and the reaction was then incubated for 18 h at 30°C. After a 2-min incubation at 65°C, the reaction was cooled to 37°C and digested with Bam HI (Thermo Fisher Scientific) for 30 min. The restriction pattern was resolved on a 0.8% agarose gel.

### Immunocytochemistry

For immunostaining of human dermal fibroblasts, the cells were cultured on coverslips and fixed at 5–7 days post electroporation in 4% paraformaldehyde (PFA)/PBS at 4°C for 15 min. Following this, the cells were washed in PBS, permeabilized in 0.5% Triton X-100/PBS for 30 min at room temperature, and blocked in 10% normal goat serum (NGS)/0.3% Triton X-100/PBS solution for 1 h at room temperature. Antigen retrieval was performed using sodium citrate solution. Coverslips were then incubated with anti-usherin (1:200) overnight at 4°C, followed by three washes in 0.1% PBST and further incubation with Alexa Fluor secondary antibody diluted 1:500 (Thermo Fisher Scientific) for 1 h at room temperature. After several washes in 0.2% PBST, the coverslips were mounted and counterstained using Prolong Diamond Antifade mountant + DAPI (Thermo Fisher Scientific). The slides were imaged using a Carl Zeiss Invert 880 microscope with Airyscan.

### Retinal immunostaining

Whole zebrafish larvae or enucleated eyes from fish 1 mpf or older were fixed in 4% PFA/PBS overnight at 4°C before washing in PBS and incubation in 30% sucrose/PBS overnight at 4°C. The samples were mounted and frozen in TissueTek O.C.T. (VWR) using dry ice. 12-μm sections were cut and collected onto Superfrost PLUS slides (Thermo Fisher Scientific). After air-drying, sections were washed in 0.5% Triton X-100/PBS before being blocked for 1 h with 20% NGS (Sigma-Aldrich) in 0.5% Triton X-100/PBS and incubation with primary antibody diluted in antibody solution (2% NGS in 0.5% Triton X-100/PBS) at 4°C overnight. The following primary antibodies and dilutions were used in this study: rabbit anti-turboGFP (Evrogen, 1:500) rabbit anti-whrna (Novus Biologicals, 1:300, kindly gifted by Dr. Jennifer Phillips, University of Oregon), rabbit anti-whrnb (Novus Biologicals, 1:300, gifted by Dr. Jennifer Phillips), and rabbit anti-adgrv1 (1:1000, gifted by Dr. Jennifer Phillips). For anti-whrna, anti-whrnb, and anti-adgrv1, antigen retrieval using sodium citrate solution was performed on slides before staining. After washing with 0.5% Triton X-/PBS, the sections were incubated with Alexa Fluor 488, 568, or 647 secondary antibody (Thermo Fisher Scientific) diluted 1:500 in antibody solution for 2 h at room temperature. Finally, the sections were washed and mounted in Prolong Diamond Antifade mountant + DAPI (Thermo Fisher Scientific). The slides were imaged using a Carl Zeiss LSM710 upright confocal microscope or Carl Zeiss Invert 880 microscope with Airyscan.

### Statistics

Data are shown as mean values ± standard deviation from n observations. The Shapiro-Wilk test was used to test for normal distribution. Student’s t tests or Mann-Whitney U tests were used to compare data. p < 0.05 was accepted to indicate statistical significance. GraphPad Prism software was used for statistical analysis.

## Data Availability

All data generated or analyzed during this study are included in this published article and its supplementary material or available from the corresponding author upon reasonable request.

## Supplemental information

Document S1. Figures S1–S6 and Table S1

Document S2. Article plus supplemental information
