# Supplementary material for: Successful large gene augmentation of USH2A with non-viral episomal vectors
Source: Mol Ther. 2023 Jun 19;31(9):2755–66. doi: 10.1016/j.ymthe.2023.06.012 (PMC10491995; doi:10.1016/j.ymthe.2023.06.012)
Supplement: Document S1. Figures S1–S6 and Table S1 [file mmc1.pdf]

YMTHE, Volume *31*

Supplemental Information

Successful large gene augmentation of *USH2A* with non-viral episomal vectors

Maria Toms, Lyes Toualbi, Patrick V. Almeida, Richard Harbottle, and Mariya Moosajee

Table S1. Primer sequences

**Primer name**	**Sequence (5'-3')**
USH2A qRT-PCR fw<br><br>*USH2A* qRT-PCR rv	GCTTGGACACCACCCTCTAC<br><br>CCCTGGGAGTAGGTTAGGCT
copGFP qRT-PCR fw<br><br>*copGFP* qRT-PCR rv	GCCGCATGACCAACAAGATG<br><br>GTTGCTGTGCAGCTCCTCCA
GAPDH qRT-PCR fw<br><br>*GAPDH* qRT-PCR rv	GCTGCATTCGCCCTCTTA<br><br>GAGGCTCCTCCAGAATATGTGA
USH2A RT-PCR fw<br><br>*USH2A* RT-PCR rv	CTGTTGCACACTGCTAAATCCC<br><br>GGTGACCCCTGAGGATCAAAAA
ef1α RT-PCR fw<br><br>*ef1α* RT-PCR rv	CAAGGGCTCCTTCAAGTACGCCTG<br><br>GGCAGAATGGCATCAAGGGCA

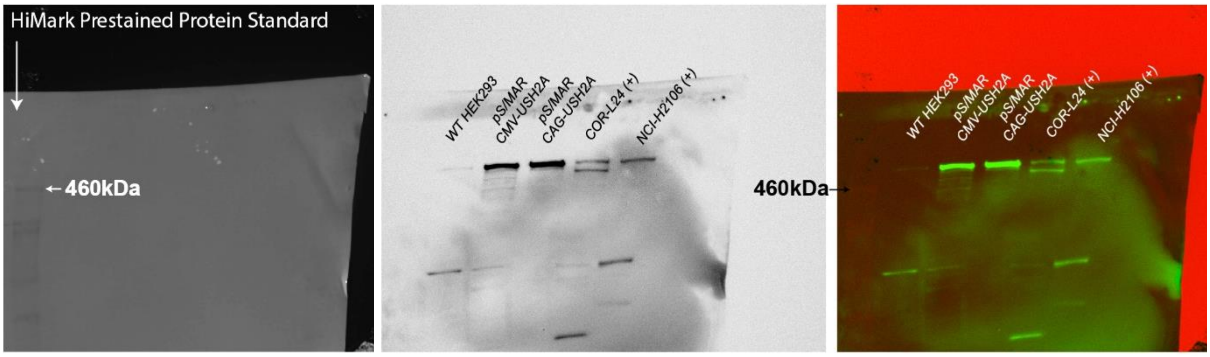

Figure S1. Full blot images for Figure 2.
Western blot for usherin performed on non-transfected (WT) HEK-293 cells, pS/MAR-CMV-USH2A and pS/MAR-CAG-USH2A transfected HEK-293 cells. Two cancer lines, COR-L24 and NCI-H2106, were used as positive controls.

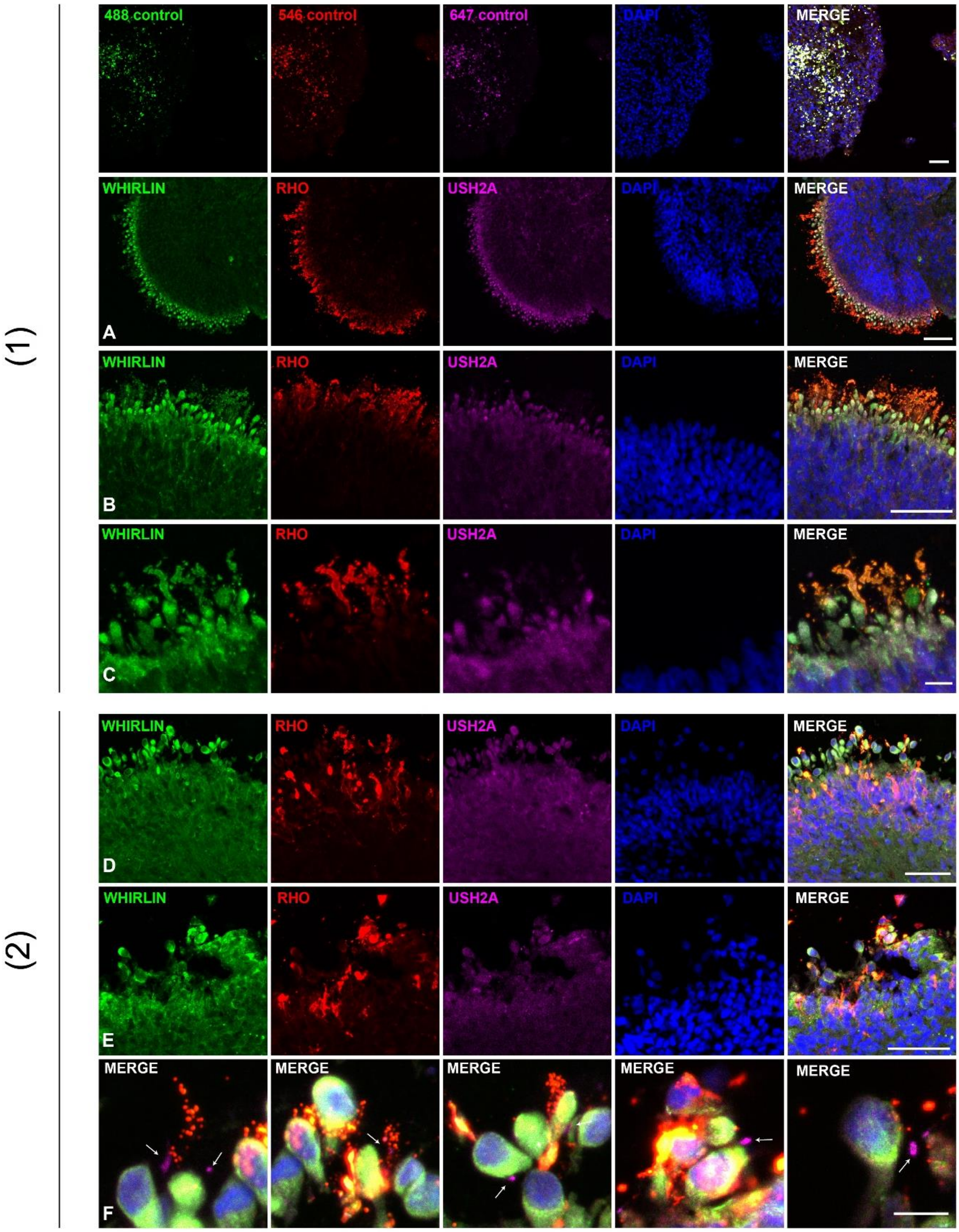

Figure S2. 42-week-old wild-type retinal organoids immunostained with anti-usherin. Two 42-week retinal organoids with preserved lamination were antibody stained for usherin (magenta), whirlin (green) and rhodopsin (red). Arrows highlight spot-like usherin signal in the photoreceptor cells. Secondary antibody-only controls are shown in the top panel. Scale bars = 50 µm (A, B, D, E) and 10 µm (C, F).

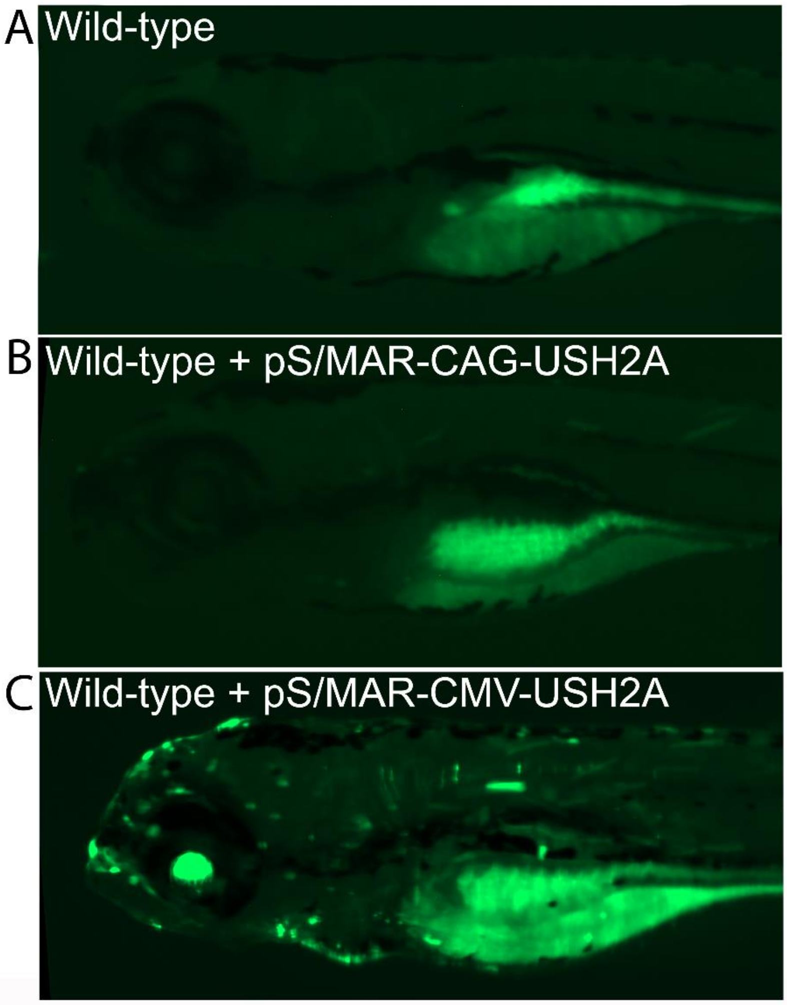

Figure S3. Comparison of USH2A-S/MAR vector expression in wild-type zebrafish. Fluorescent microscope images showing GFP expression (green) in 5 days post-fertilisation wild-type zebrafish (A) un-injected or micro-injected at the one-cell stage with (B) pS/MAR-CAG-USH2A or (C) pS/MAR-CMV-USH2A.

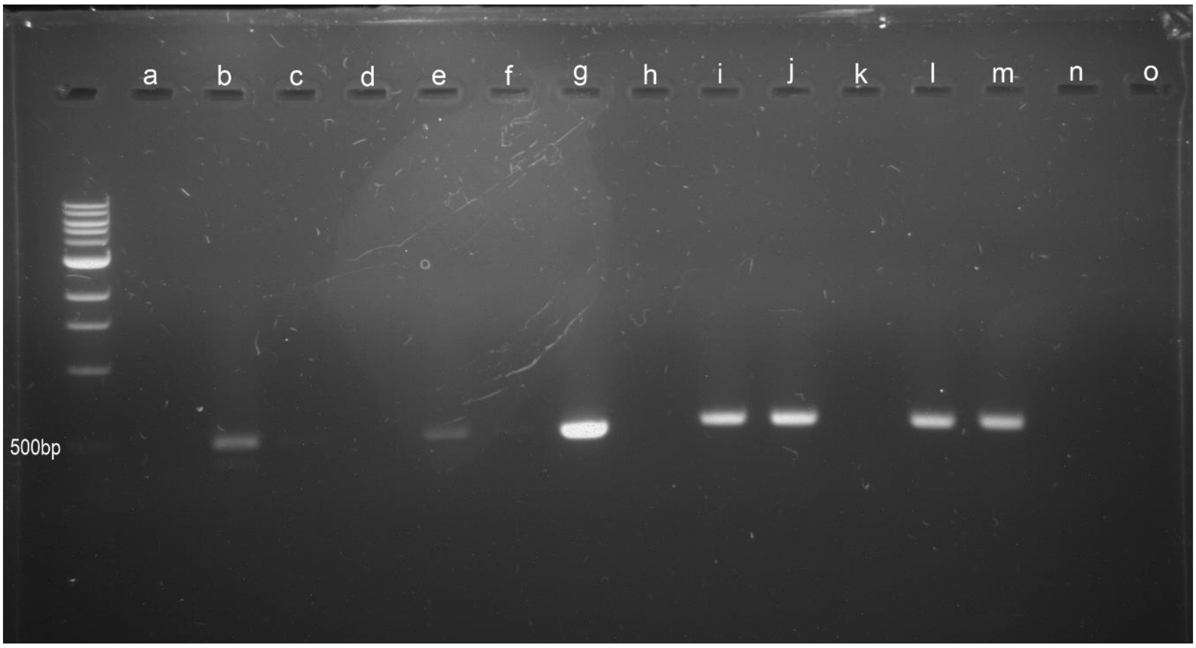

Figure S4. Full gel picture for Figure 4.
RT-PCR of human *USH2A* in 5 dpf zebrafish samples: (a) un-injected wild-type, (b) wild-type micro-injected with pS/MAR-CMV-USH2A, (c) injected wild-type no reverse transcription (no RT) control, (d) un-injected *ush2a*^{u507}, (e) *ush2a*^{u507} micro-injected with pS/MAR-CMV-USH2A, (f) injected *ush2a*^{u507} no RT control, (g) pS/MAR-CMV-USH2A plasmid positive control, (h) H_2O control. RT-PCR of *ef1α* in 5 dpf zebrafish samples: (i) un-injected wild-type, (j) wild-type micro-injected with pS/MAR-CMV-USH2A, (k) injected wild-type no RT control, (l) un-injected *ush2a*^{u507}, (m) *ush2a*^{u507} micro-injected with pS/MAR-CMV-USH2A, (n) injected *ush2a*^{u507} no RT control, (o) H_2O control.

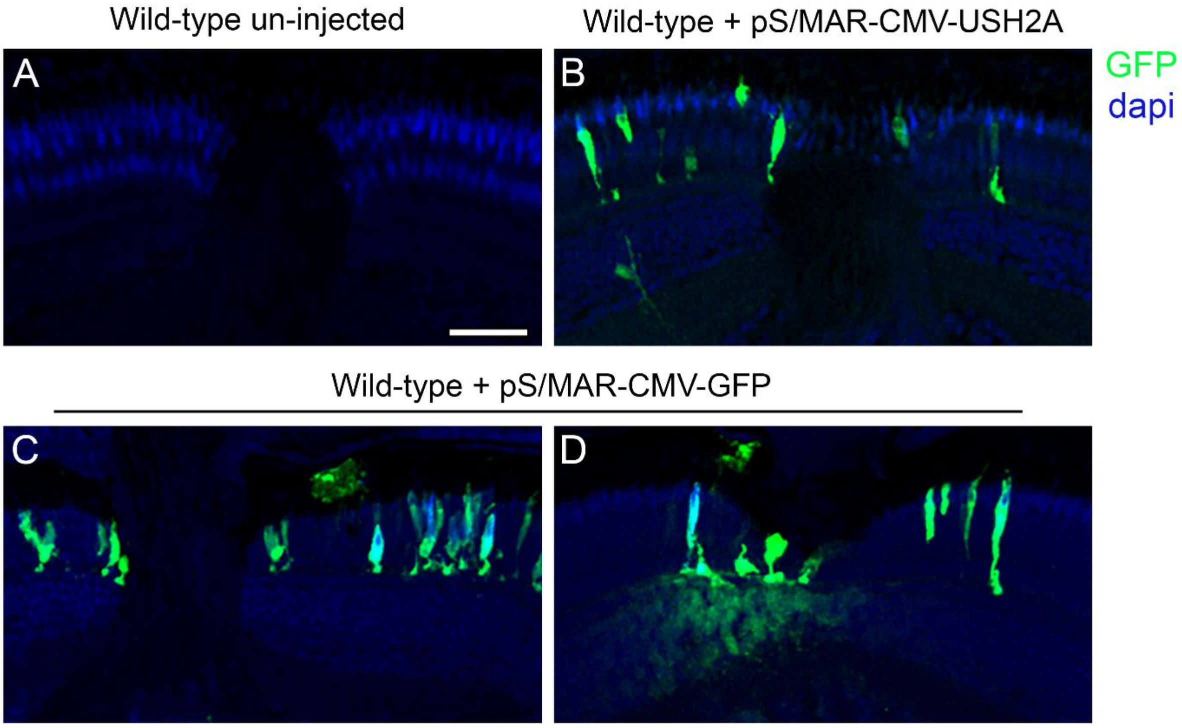

Figure S5. Comparison of 1 mpf retinas from wild-type zebrafish injected with either pS/MAR-CMV-USH2A (B) or pS/MAR-CMV-GFP (C, D). Cryosections are immunostained with anti-GFP (green) and counterstained with dapi (blue). Scale bar = 25 µm.

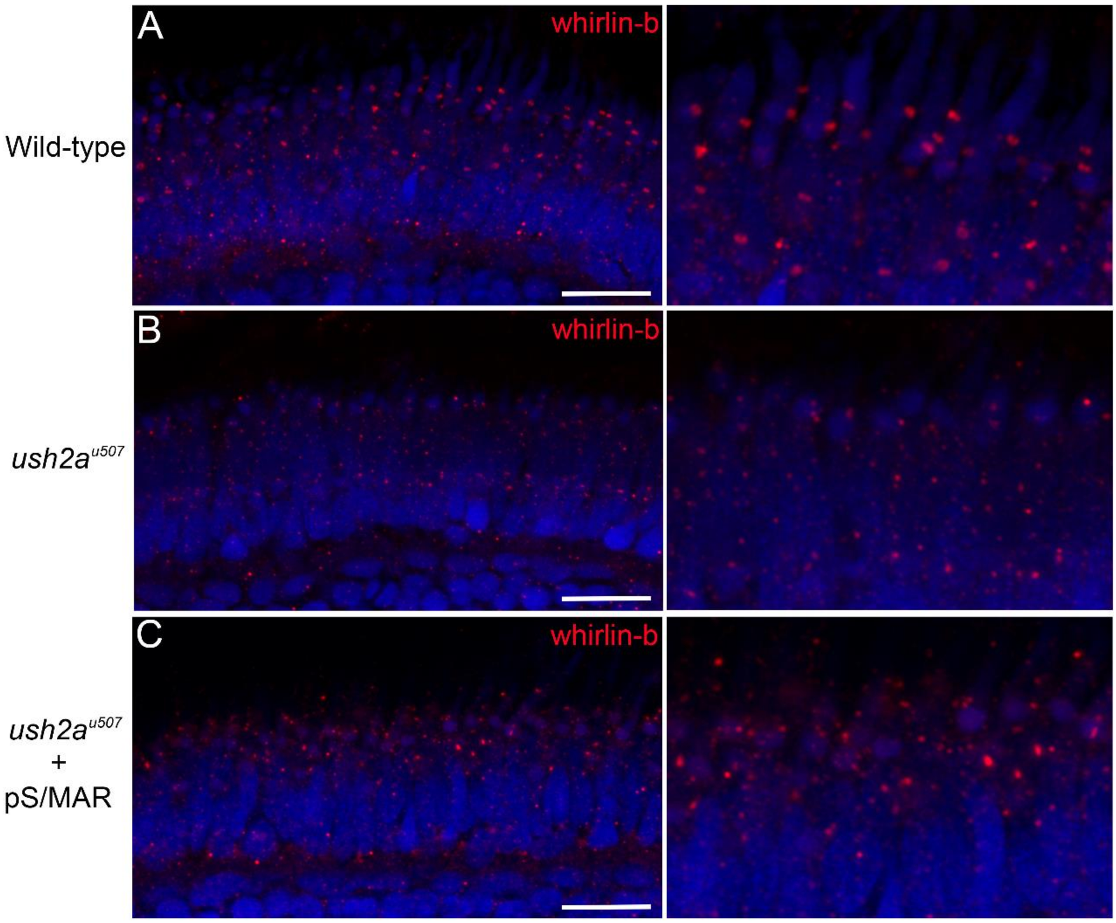

Figure S6. Partial rescue of whirlin-b expression in pS/MAR-USH2A-injected *ush2a^{u507}* zebrafish.

Retinal sections from zebrafish at 6 days post-fertilisation were immunostained for Usher 2 complex protein, whirlin-b. Specific punctate expression of whirlin-b (red) was detected in the wild-type photoreceptors (A) but was mislocalised in the *ush2a^{u507}* retina (B). In *ush2a^{u507}* zebrafish injected with pS/MAR-CMV-USH2A at the one-cell stage, specific whirlin-b expression could be observed in some photoreceptors (C). Scale bar = 10 µm.
